# The Influence of Thermoplastic Composite Recycling on the Additive Manufacturing Process and In-Use Phase as Candidate Materials for Wearable Devices Applications

**DOI:** 10.3390/polym15183775

**Published:** 2023-09-15

**Authors:** Alexandra Papatheodorou, Iakovos Gavalas, Despoina Ntenekou, Anna Karatza

**Affiliations:** BioG3D P.C., 1 Lavriou Ave., Technological & Cultural Park of Lavrion, 19500 Lavrion, Greece; apapatheodorou@biog3d.gr (A.P.); igavalas@biog3d.gr (I.G.); dntenekou@biog3d.gr (D.N.)

**Keywords:** printability evaluation, process parameters optimization, composite materials, recycled 3D-printing filaments, cytotoxicity

## Abstract

Fused filament fabrication (FFF) is a popular additive manufacturing (AM) method for creating thermoplastic parts with intricate geometrical designs. Pure thermoplastic materials utilized in FFF, whose polymeric matrix is reinforced with other materials, such as carbon fibers (CFs), introduce products with advanced mechanical properties. However, since not all of these materials are biodegradable, the need for recycling and reuse immediately emerges to address the significant problem of how to dispose of their waste. The proposed study evaluates the printability, surface morphology and in vitro toxicity of two thermoplastic-based composite materials commonly used in wearable device manufacturing to provide enhanced properties and functionalities, making them suitable for various applications in the field of wearable devices. Tritan Copolyester TX1501 with 7.3% chopped CFs (cCFs) and Polyamide 12 (PA12) with 8.6%cCFs and 7.5% iron Magnetic Nanoparticles (MNPs)—Fe_4_O_3_ were used in the discrete ascending cycles of recycling, focusing on the surface quality performance optimization of the printed parts. Through stereoscopy evaluation, under-extrusion, and over-extrusion defects, as well as non-uniform material flow, are assessed in order to first investigate the influence of various process parameters’ application on the printing quality of each material and, second, to analyze the optimal value fluctuation of the printing parameters throughout the recycling cycles of the materials. The results indicate that after applying certain adjustments to the main printing parameter values, the examined recycled reinforced materials are still effectively 3D printed even after multiple cycles of recycling. A morphology examination using scanning electron microscope (SEM) revealed surface alterations, while a cytotoxicity assessment revealed the adverse effects of both materials in the form of cell viability and the release of proinflammatory cytokines in the cell culture medium.

## 1. Introduction

The application of recycled composite materials in 3D printing has gained significant attention in recent years due to its potential to reduce material waste and environmental impact while generating unique, custom parts and endeavoring to retain their performance requirements. The influence of composite materials on the mechanical properties of additively manufactured parts has been studied by the research community with respect to the correlation between materials and printing settings—such as fiber concentrations, printing direction and infill ratios—with the acquired mechanical characteristics [1,2], as well as with respect to the comparison between fiber-reinforced and pure thermoplastic materials [3,4]. Moreover, methodologies integrating recycling and FFF manufacturing from scraps of wind-turbine blade components have been suggested, including investigations into the mechanical characteristics, the internal microstructure and interface of specimens utilizing recycled glass fibers (rGFs), and PLA [5,6]. According to other studies, glass-fiber-reinforced polypropylene (GFRPP) [7] and basalt-reinforced polypropylene (PP) [8] composite waste was converted to short-fiber filament, and the effect of the fibers’ weight fraction on the material properties of the obtained 3D-printed specimens was studied. Additionally, comparisons between pure polylactic acid (PLA), PLA with recycled carbon fibers (rCFs) and PLA with virgin carbon fibers (vCFs) of the same concentration were performed, and the materials were compared in terms of thermal and mechanical properties, indicating comparable thermo-mechanical performance between rCF and vCF applications [9].

The sustainability and growth of the circular economy have generally received significant attention in the FFF sector. Therefore, [10,11] literature reviews present a detailed overview of research carried out on FFF polymer-based materials, where a comparison between recycled and respective virgin filaments is performed and recent advances in innovative polymer-based materials are introduced, respectively. Moreover, an analysis on distributed recycling via an additive manufacturing (DRAM) chain is proposed, comprising six stages, recovery, preparation, compounding, feedstock, printing and quality, integrating and indicating future study approaches at the micro, meso, and macro level to better comprehend the trade-offs between a circular economy and effectively distributing recycling [12]. Another interesting approach is the development and physical, thermal and mechanical characterization of innovative composite filaments made from PLA and different olive-wood scrap compositions presented in [13], where a life cycle assessment (LCA) is also performed to designate the environmental impact indicators. Furthermore, although the recycling of thermoplastics commonly used in FFF, namely PLA, poly(ethylene terephthalate) (PET), PP and high-density polyethylene (HDPE), has attracted great interest in terms of recycling procedure optimization, including the determination of virgin-to-recycled-material composition, the extrusion parameters and type of extrusion, as well as the characterization of the obtained 3D-printed specimens [14,15,16,17], based on our literature review, comparable studies on PA12 or TX1501 with CF additives are not available. A research study [18] investigated the utilization of three different recycled PA12 polymers obtained as residuals from the selective laser sintering process. Through mechanical assessments, direct comparisons of recycled PA12, PA12 reinforced with 5 wt% aramid fibers and recycled PA12 incorporated with 10 wt% polyurethane to virgin pure PA12, were conducted. The findings revealed a reduction in tensile strength when employing recycled PA12. In contrast, the values of the tensile strength were comparable to pure PA12 when the PA12 was reinforced with 5 wt% aramid fibers. However, the introduction of 10% polyurethane to virgin PA12 resulted in tensile strength enhancement relative to virgin pure PA12.

Aside from the physical, mechanical and thermal characterization of a filament utilized for FFF manufacturing, the correlation between the applied process parameters and printability performance plays a key role in material assessment, and therefore, great focus is given to printing parameter optimization for defect moderation [19,20]. Benchmark geometries and process-specific phenomena, like the stair-stepping effect, have been applied to assess the dimensional accuracy and manufacturability constraints of reinforced nylon with cCFs, where the SEM observation of the manufactured parts illustrated a significant degree of mechanical property anisotropy due to inadequate interlayer adhesion in the build direction [3]. Moreover, a study on the influence of the 3D-printing parameter on the geometric behavior of PET-G with CF filament, employing artificial-neural-network-based predictive models for the optimization of printing parameters, indicates that the addition of CFs diminishes both the number of printing parameter combinations, where the optimal values are managed, and the dimensional accuracy and surface quality of the manufactured parts in most of the printing conditions [21].

To the best of our knowledge, the current study introduces a novel investigation into the printability and cytotoxicity performance across multiple recycling cycles of custom CF-reinforced PA12- and TX1501-based materials, which are designed for applications in wearable devices, while suggesting a fast material screening and evaluation workflow. More specifically, a connection between recycling iterations and printability behavior is investigated on materials developed within the frame of the European research project Repair3D-Recycling and Repurposing of Plastic Waste for Advanced 3D Printing Applications (Grant Agreement no. 814588). Thus, three distinct cycles of PA12 with 8.6%cCFs and 7.5% iron MNPs and TX1501 with 7.3%cCFs are evaluated and compared to the respective initially developed material batches in order to check the influence of recycling in the printing process, to determine the optimal process parameters, and to observe the value fluctuation and surface variation. The printability evaluation of discrete recycling cycles enables the depiction of the material efficiency variations during FFF, as well as the determination of the adjusted process parameter values to optimize printing performance and manage the highest-quality output with every recycling cycle. Thus, a straightforward workflow for material evaluation and printing optimization of different recycling cycles is established based on material performance, as observed through stereoscope inspection instead of extended material testing and expensive characterization methods. Moreover, as a second stage of the material printability study, SEM morphology assessment was conducted to examine the impact of each recycling cycle on the printing process parameters in comparison to the established optimal process parameters of the control material. Additionally, since the recycled materials evaluated in the present study are to be used in the development of wearable devices, cytotoxicity studies were implemented to evaluate the material’s effects after coming in direct contact with human cells.

TX1501 with 7.3%cCFs and PA12 with 8.6%cCFs and 7.5% iron MNPs were appointed to the manufacturing of a wrist hand orthotic device and a ski boot, respectively. As both wearable devices require advanced mechanical properties and high printing performance for good ergonomic fit, CFs were introduced, and printing optimization was established to meet these specifications, respectively. Furthermore, wearable devices come with various applications that cater to different needs and user preferences, such as health, fitness and medical monitoring. To enhance the commercial applications of these wearable devices, several additional properties can be considered. For medical and health-focused wearables, compliance with relevant healthcare regulations and standards is crucial for commercial success and user trust. Except for the hazard assessment of the materials used in wearable devices, as described in the current study, the antimicrobial properties of the materials are also crucial for their safe use. Furthermore, leaching behavior of the nanocomposite materials in solutions that simulate washing and/ or use conditions, such as water or sweat, respectively can provide information necessary to mitigate any potential risks associated with the release of additives and their skin-sensitizing potential, ensuring the development of wearable devices that are both functional and safe for user [22].

## 2. Materials and Methods

### 2.1. Materials

Printability evaluation tests were performed on two reinforced thermoplastic materials: TX1501 with 7.3%cCFs and PA12 with 8.6%cCFs and 7.5% iron MNPs. TX1501 and PA12 matrices of the composites were provided by Eastman (Eastman Chemical B.V, Watermanweg, The Netherlands) and Grilamid EMS (EMS-CHEMIE AG, Domat/Ems, Switzerland), respectively, while the incorporated CFs originated from Sigmatex (Sigmatex Europe, Cheshire, UK) and have been cut to a length of 6mm. The PA12-based composite was also enriched with iron MNPs 99.5% purity, 20 nm in diameter, 1% PVP-coated, and supplied by GetNanoMaterials (Oocap SAS, Saint Cannat, France). The initially developed composites, called Control Material, and the filament produced after the first, fifth and tenth recycling cycles were subjected to printability and cytotoxicity assessment. The implemented printability tests intended to study the fluctuation of the optimal main printing parameters, namely printing speed, extrusion temperature, track height and track width, between Control Material and recycled filaments, while a cytotoxicity assessment was performed to reveal possible adverse effects of the recycled materials after coming in direct contact with human cells.

Material selection was based on the requirements of the final 3D-printed products. The PA thermoplastic matrix was chosen due to its strong adhesion with textiles for the production of 3D-printed wearable devices [23] with pertinent specifications while providing the required stiffness at the same time. Additionally, MNPs were added into the thermoplastic matrix to offer either self-healing properties that can repair minor damages, like scratches or cracks [24], or heating induction [25] in order to advance the durability of the final ski boot. Moreover, iron MNPs have also been investigated as additives for 3D-printed magnets and soft-robotics applications [26]. Concerning the wrist hand orthosis parts [27], TX1501 was chosen as a potential matrix, incorporating cCFs [28,29] to improve the mechanical properties of the final manufactured products. In addition, the 3D printing of thermoplastic composite filaments with CFs have been extensively investigated in multiple industrial sectors, such as aerospace, defense and automotive, due to their mechanical and thermal properties [30].

### 2.2. Workflow and Methods

**Figure 1:**
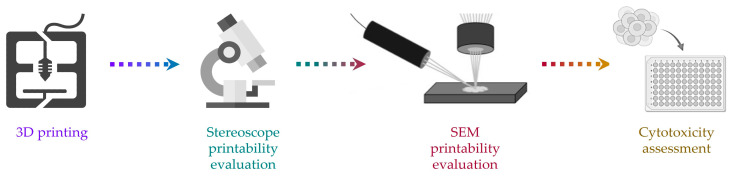
Workflow of the evaluation of recycled 3D printing materials.

Figure 1 illustrates the workflow followed in the presented study, consisting of four stages: 3D printing, printability evaluation with stereoscope, printability evaluation with SEM, and cytotoxicity assessment. The following paragraphs present the methods developed for the realization of printability and cytotoxicity assessment in detail.

#### 2.2.1. Printability Assessment

In order to compare the fluctuation of the optimal printing parameters between the recycling cycles, the values combinations examined for each material were determined according to the printability ranges of the Control Material and were consistent for all tested cycles. Customized toolpaths based on the literature [31] were generated using FullControl GCODE Designer Heron02d software (https://fullcontrolgcode.com/ (Accessed: 4 September 2023)) to perform interdependent parametrization tests. The test samples were manufactured using a Raised3D Pro2 Plus 3D (Raise3D, Irvine, CA, USA) printer, whose build volume is 305 × 305 × 605 mm under STP conditions.

**Figure 2:**
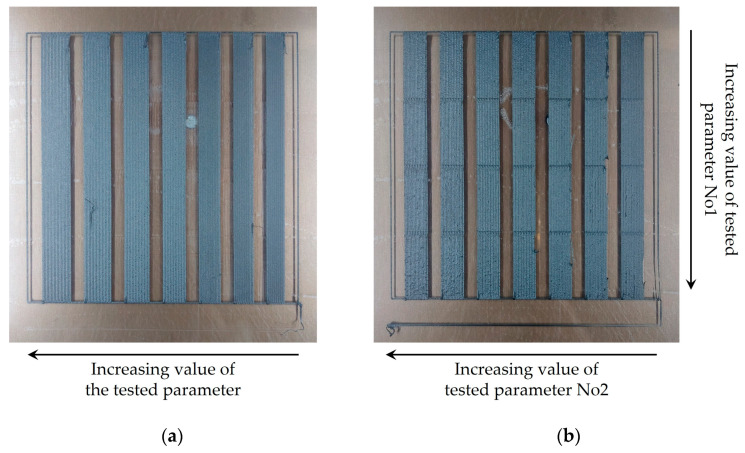
Samples of TX1501 with 7.3%cCFs printability tests: (**a**) printing sample with one varying process parameter; (**b**) printing sample with two varying process parameters.

The first stage of printability evaluation was performed using a Leica S9D stereoscope (Leica Microsystems, Wetzlar, Germany). Two types of printability samples were generated by either testing (a) one single printing parameter or (b) two printing parameters at the same time. In the first case, the tested parameter value increases while moving horizontally from each printed region, whereas in case of the two parameters examined, the one parameter value increases while moving horizontally while the other increases while moving downwards, as illustrated in Figure 2. Table 1 displays all combinations of printing parameters examined at each test.

The visibly unacceptable regions on the printed parts, for example, unfinished or unprinted regions, are immediately rejected, while the remaining regions are examined using a stereoscope to detect defects due to under- or over-extrusion and assess the uniformity of the material flow for the selection of the region with the optimal printing parameters. In case there are multiple regions without defects, the optimal region is determined as the one that combines the more efficient printing parameters, namely greater printing speed, layer height or layer width.

**Figure 3:**
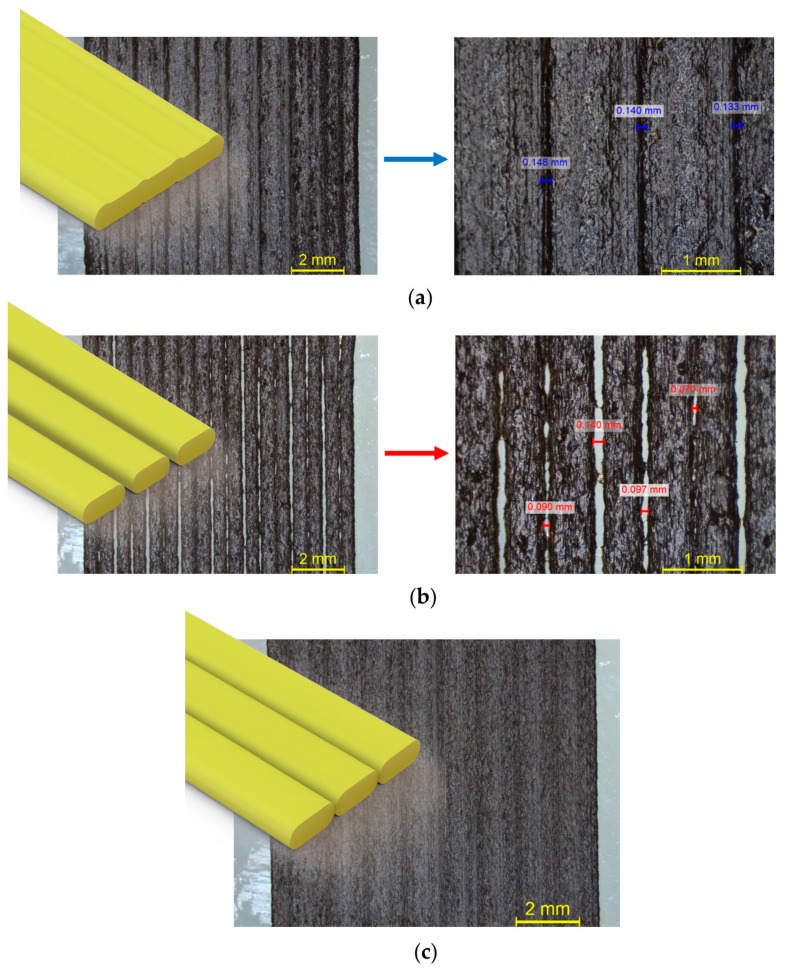
Examples of printing track types observed with the stereoscope: (**a**) over-extrusion; (**b**) under-extrusion; (**c**) targeted track overlap.

**Figure 4:**
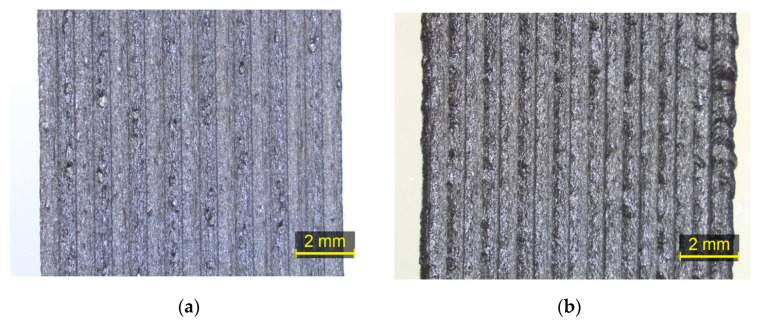
Examples of regions with different material flow uniformity: (**a**) region with high uniformity; (**b**) region with low uniformity.

Figure 3 shows examples of printing track types that can be observed with the stereoscope, namely over-extrusion, under-extrusion, and the accepted track overlap, utilizing two different zoom scale levels for the defective regions. The first scale value is applied as a preliminary inspection of the tested region for the presence of over- or under-extrusion defects, while the focused scale facilitates the quantification of the presented defects, where over-extrusion and under-extrusion defects are annotated in blue and red, respectively. Figure 4 exhibits two regions with different levels of material flow uniformity.

**Figure 5:**
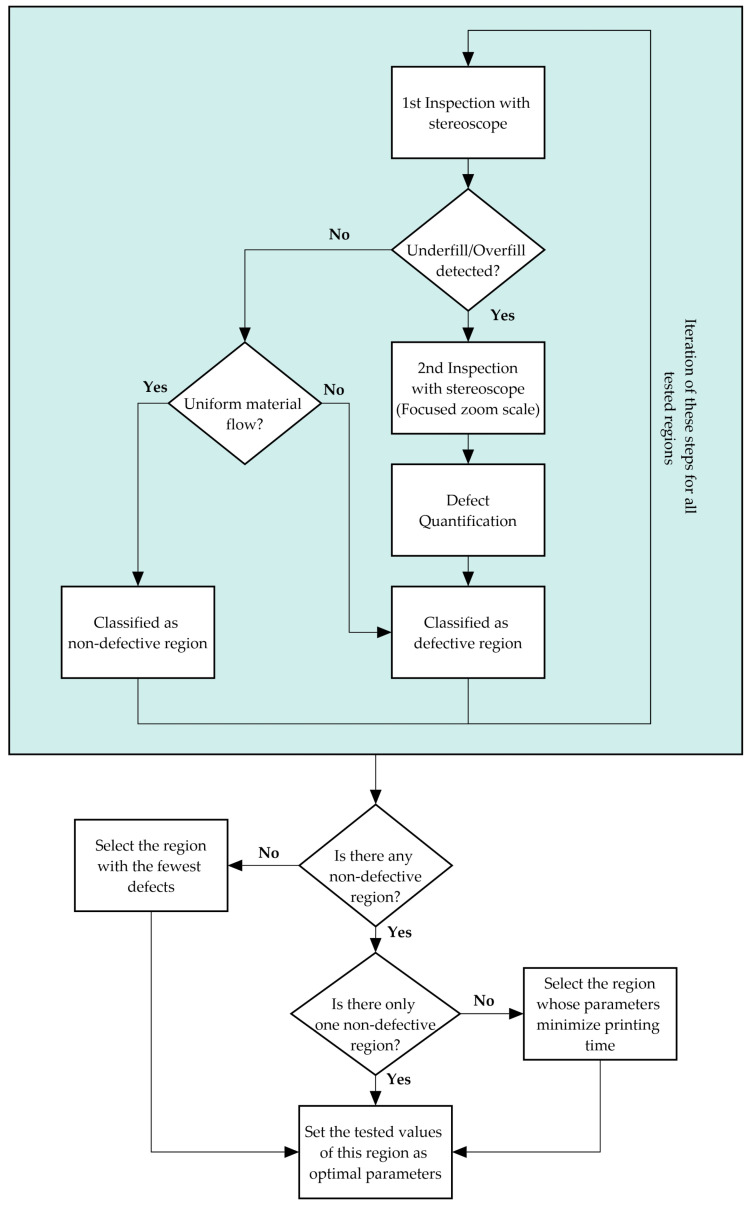
Stereoscope printability evaluation workflow.

Figure 5 illustrates the workflow followed during the stereoscope printability evaluation of each test for the determination of the optimal printing parameters.

The second stage of printability assessment was utilized using a desktop SEM (Phenom ProX, ThermoScientific), and the analysis was conducted by comparing a part of the Test01 regions of every recycling cycle corresponding to the optimal First-layer track height and printing speed selected for the Control Material of each material. The other printing parameters applied are displayed in Table 2. The remnants of these regions were subjected to cytotoxicity assessment. Moreover, in order to further assist the SEM morphology assessment, surface roughness evaluation was conducted by measuring the grey values of the SEM images. Using the open source software ImageJ 1.53t (https://imagej.net/software/imagej/ (Accessed: 4 September 2023)), the grey values were obtained alongside the length of the diagonal line which, connects the left bottom corner with the right top corner of each image. The raw data was then post-processed and smoothed using the Adjacent-Averaging method. This surface evaluation approach is an indirect analysis to study surface roughness, and it can only be used for qualitative assessment since no data from the z axis can be obtained.

#### 2.2.2. In Vitro Toxicity Assessment of the Recycled Materials

Before conducting in vitro testing, material samples underwent sterilization by immersion in 70% ethanol (*v*/*v*) overnight. Then, they were rinsed three times with 1x PBS to eliminate all traces of ethanol. Subsequently, the samples were air-dried in a sterile atmosphere and sterilized for 1 h using ultraviolet light. This process ensured the removal of any surface pollutants to prevent contamination [32]. Upon microscopy evaluation, no microbial contamination was observed in any of the samples during the experiment.

After sterilization and prior to testing, the samples were immersed in complete DMEM overnight. Human skin fibroblasts (Hs27, CRL-1634), obtained from foreskin and complying with the ISO 10993-5 standard for cytotoxicity testing, were purchased from the American Type Culture Collection (ATCC) located in Manassas, VA, USA. The cells were cultured to confluence in complete DMEM (PAN-Biotech, Aidenbach. Germany, Cat. No. P04-03590) supplemented with 10% FBS (PAN-Biotech, Aidenbach. Germany, Cat. No. P30-1985) and 1% penicillin/streptomycin (BIOSERA, Cholet, France Cat. No. XC-A4122) solution. The incubation of cells took place at 37 °C in a CO_2_ incubator (Thermo Fisher Scientific, Waltham, MA USA) with 5% carbon dioxide. The sterilized samples were placed separately in 12-well plates, followed by direct cell seeding at a density of 1 × 50^4^ cells per well. A tissue culture plate containing fresh DMEM and only cells was used as a positive control. After 48 h of exposure, the cell culture medium was analyzed using ELISA, following the manufacturer’s protocols, to measure the release of the proinflammatory cytokines IL-18 (OriGene, Rockville, USA, Cat. No EA100549) and TNF-α (AssayGenie, Dublin, Ireland, Cat. No. HUFI00262) as indicators of potential skin sensitization. Furthermore, cell viability upon exposure for 48 h to the recycled materials was also measured using the MTT assay and a microplate reader (FLUOstar omega, BMG Labtech, Ortenberg, Germany). The MTT (Sigma-Aldrich, Misouri, USA Cat. No. 475989) solution was made at a concentration of 5 mg/mL, and 1/10 of the total well plate volume was placed in each well.

## 3. Results

### 3.1. Printability Assessment

#### 3.1.1. PA12 8.6%cCFs and 7.5%MNPs

The printability tests on all examined cycles of PA12 with 8.6%cCFs and 7.5%MNPs were realized utilizing the same printing parameter values within the printable ranges of the material. Table 3 presents the selected process parameter values, and since the printing speed of the main layers is examined three times in order to be fine-tuned, the values of this parameter in Test04 and Test06 were defined for each recycling cycle independently. The printing speed values applied in Test04 were deduced from the optimal value derived from Test03, introducing a 10 mm/s step between the tested values. Accordingly, the printing speed values of Test06 were set based on the optimal printing speed value of Test04 and by applying a 2.5 mm/s step. Table 4 presents the printing speed values examined in Tests04 and Tests06, and the corresponding optimum values selected in each case of recycling cycle of PA12 with 8.6%cCFs and 7.5%MNPsevaluated.

**Figure 6:**
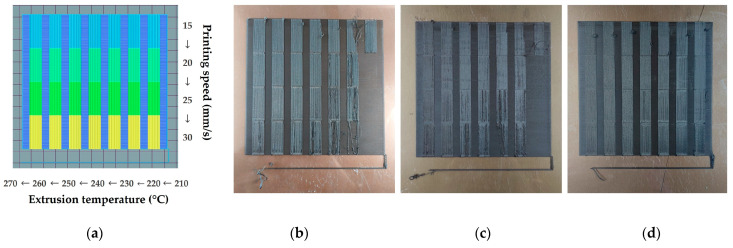
Temperature vs. Printing speed in Test03 samples of PA12 with 8.6%cCFs and 7.5%MNPs: (**a**) printing parameters model image; (**b**) Control Material sample; (**c**) 1st Cycle sample; (**d**) 5th Cycle sample.

**Figure A1:**
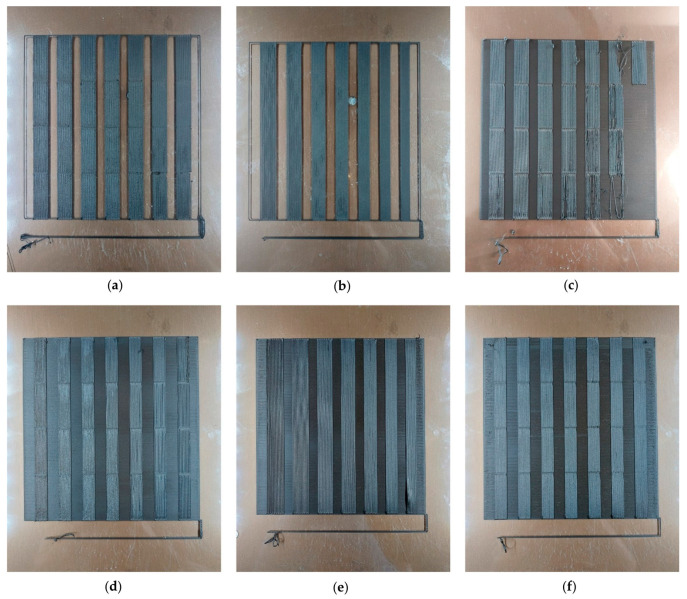
Printability samples of PA12 with 8.6%cCFs and 7.5%MNPs (Control Material): (**a**) Test01; (**b**) Test02; (**c**) Test03; (**d**) Test04; (**e**) Test05; (**f**) Test06.

**Figure A2:**
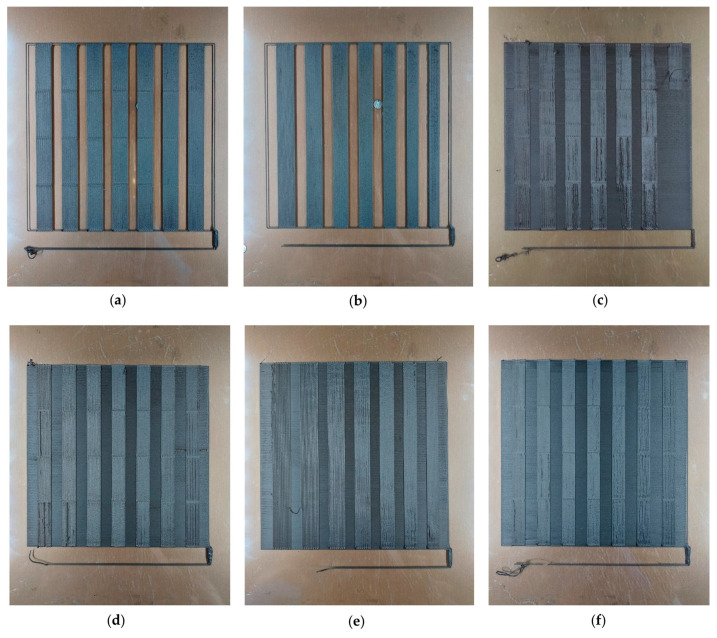
Printability samples of PA12 with 8.6%cCFs and 7.5%MNPs (1st Cycle): (**a**) Test01; (**b**) Test02; (**c**) Test03; (**d**) Test04; (**e**) Test05; (**f**) Test06.

**Figure A3:**
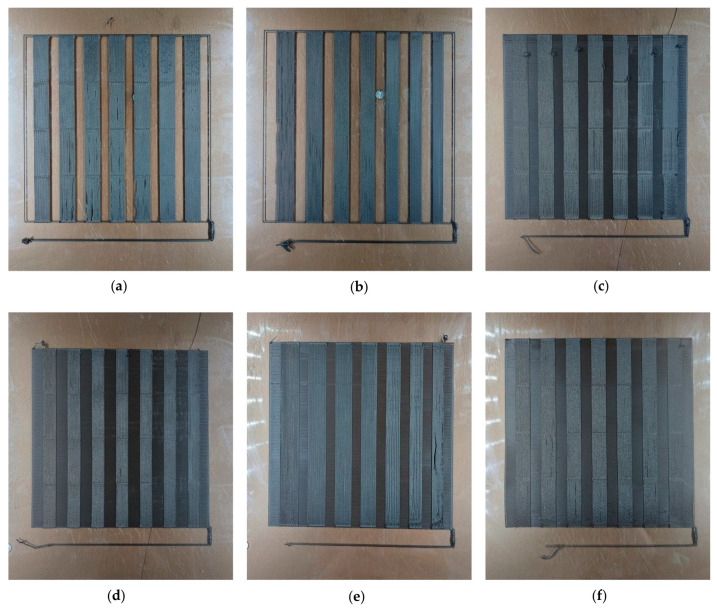
Printability samples of PA12 with 8.6%cCFs and 7.5%MNPs (5th Cycle): (**a**) Test01; (**b**) Test02; (**c**) Test03; (**d**) Test04; (**e**) Test05; (**f**) Test06.

**Figure A4:**
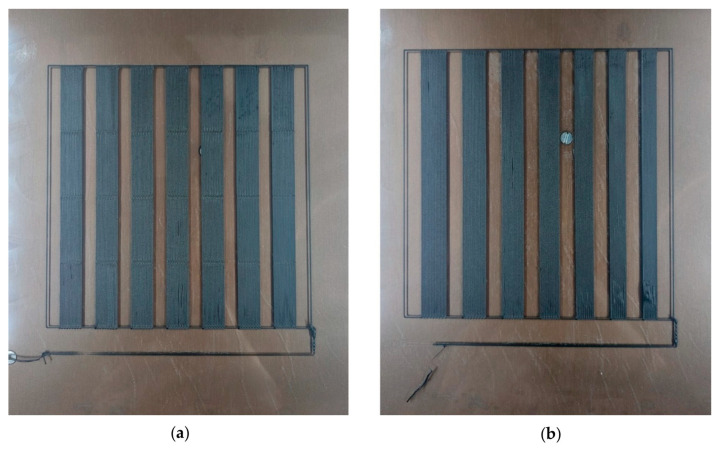
Printability samples of PA12 with 8.6%cCFs and 7.5%MNPs (10th Cycle): (**a**) Test01; (**b**) Test02.

**Figure A5:**
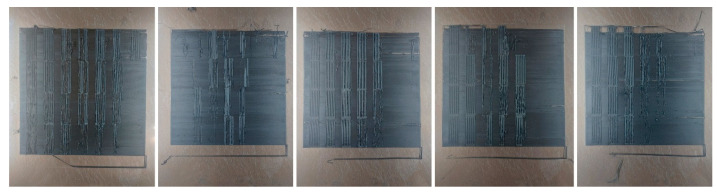
Different printing samples of Test03 PA12 with 8.6%cCFs and 7.5%MNPs (10th Cycle).

Figure A1, Figure A2, Figure A3, Figure A4 and Figure A5 present the printability samples produced with the Control Material, 1st Cycle, 5th Cycle and 10th Cycle of PA12 with 8.6%cCFs and 7.5%MNPs. As can be deduced from Figure A1 and Figure A2, the Control Material and 1st Cycle of PA12 with 8.6%cCFs and 7.5%MNPs presented satisfactory printability, except for printing temperature values below 230 °C (Figure 6b,c). On the contrary, the 5th Cycle of PA12 with 8.6%cCFs and 7.5%MNPs was printable at all parameter values tested, even when utilizing printing temperatures in the range of 210–230 °C (Figure 6d). The printability samples illustrated in Figure A4 were realized for the determination of the First-layer process parameters, whereas the samples presented in Figure A5 correspond to the discrete printing attempts of Test03. However, due to multiple nozzle clogging issues and inadequate adhesion, both between the printing layers and between the first layer and the print bed surface, the 10th Cycle was classified as non-printable.

**Figure 7:**
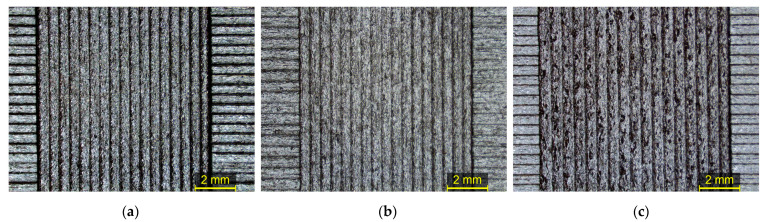
Stereoscope images of Test06 optimum regions: (**a**) Control Material; (**b**) 1st Cycle; (**c**) 5th Cycle.

Table 5 displays the optimal parameters derived from the printability assessment of the Control Material, 1st Cycle, 5th Cycle and 10th Cycle of PA12 with 8.6%cCFs and 7.5%MNPs, and Figure 7 illustrates the Test06 stereoscope images that correspond to the regions, with the optimum printing parameters of Control Material and all cycles examined as well. As the recycling cycles progress, an augmentation in printing speed is observed. This phenomenon can be explained by the gradual degradation of the polymeric matrix over recycling iterations, resulting in a reduction in the molecular weight of the polymeric chains. This reduction induces a decrease in the melting viscosity of the matrix, thereby facilitating an escalated flow rate during the extrusion process. Consequently, higher extrusion velocities are applied to overcome over-extrusion, a condition wherein an excessive amount of thermoplastic material flows from the nozzle, leading to the thickening of the track width.

**Figure 8:**
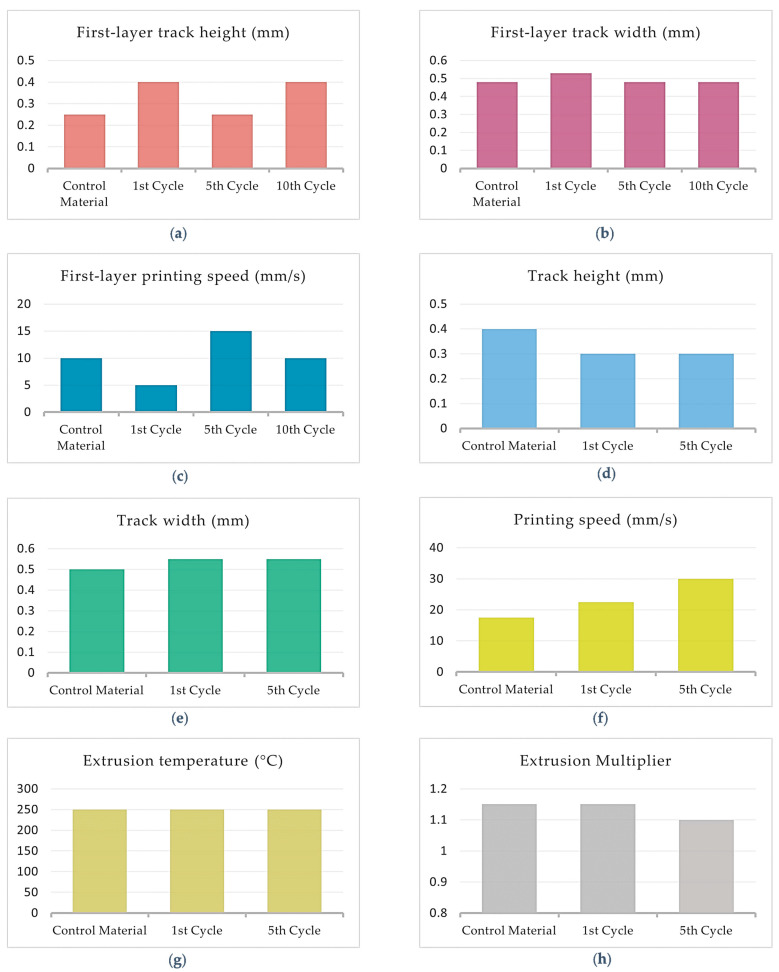
Optimal printing parameter value fluctuation of PA12 with 8.6%cCFs and 7.5%MNPs through recycling cycles: (**a**) First-layer track height; (**b**) First-layer track width; (**c**) First-layer printing speed; (**d**) Track height; (**e**) Track width; (**f**) Printing speed; (**g**) Extrusion temperature; (**h**) Extrusion multiplier.

Figure 8a–h shows the fluctuation of the optimal printing parameters of PA12 with 8.6%cCFs and 7.5%MNPs through the recycling cycles. Since the 10th Cycle filament was not printable, only the first three tests concerning the First-layer parameters were completed successfully. Therefore, Figure 8a–c display the results from all tested cycles, whereas Figure 8d–h presents the optimal values of the main layer parameters established for the Control Material, 1st Cycle and 5th Cycle.

Concerning the First-layer process parameters, the optimal Track height and printing speed values for PA12 with 8.6%cCFs and 7.5%MNPs oscillate in the ranges of 0.25–0.40 mm and 5–15 mm/s, respectively (Figure 8a–c), while the optimal track width value presents minor fluctuation through the recycling process since the maximum variation from the optimal value of the Control Material is 10.4% (Figure 8b). In regard of the main printing layers, the track height optimal value seems to converge to 0.3 mm after the recycling procedures (Figure 8d) and the track width optimal values present maximum 9.1% fluctuation from the respective value established for the Control Material. As can be inferred from Figure 8f,g, the optimal printing speed presents a gradual increase from 17.5 mm/s to 30 mm/s, while the optimal value of the extrusion temperature is not affected by the recycling process and remains constant at 250 °C. Finally, the optimal values of the extrusion multiplier for PA12 with 8.6%cCFs and 7.5%MNPs do not exhibit significant fluctuation, as the maximum variation from the respective Control Material value is 4.5% (Figure 8h).

**Figure 9:**
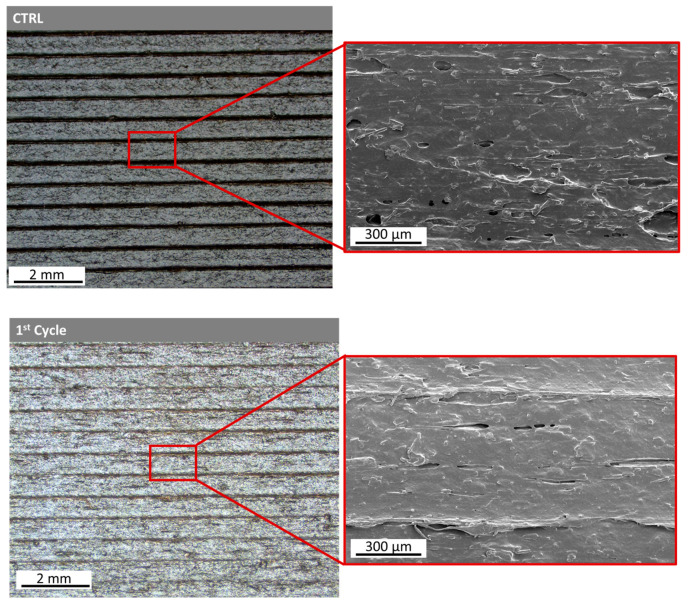

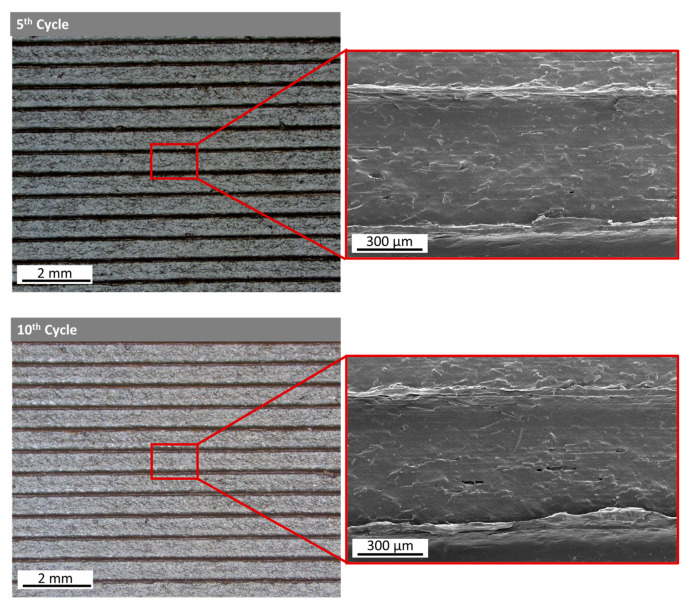
Stereoscope and SEM images for surface assessment after the first, fifth and tenth recycling cycles of nanocomposite PA12 filament. Surface comparison using the control’s optimized printing parameters in every recycling cycle.

**Figure 10:**
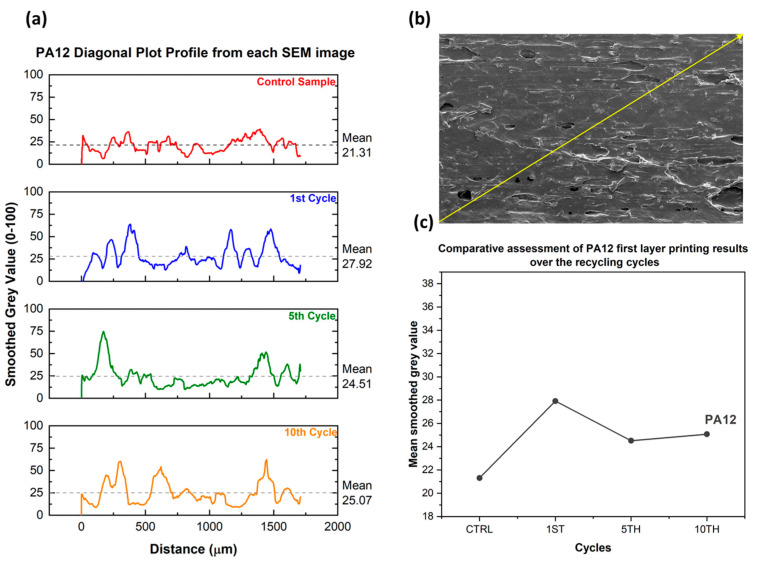
(**a**) Smoothed plot profiles from the grey values obtained alongside the diagonal line from each PA12 SEM image. (**b**) Indicative diagonal line from which the grey values were obtained. (**c**) The evolution of the mean grey values from each recycling cycle. Surface comparison using the control’s optimized printing parameters in every recycling cycle.

A surface morphology comparison using the Control Material optimized printing parameters in every recycling cycle was conducted by employing SEM morphology assessment (Figure 9). Image processing utilizing ImageJ was performed, and surface plot profiles were generated for every recycling cycle to assess the effect of the recycling cycles (Figure 10a) on the surface roughness alongside the yellow diagonal line (Figure 10b). Then, the mean grey values were plotted in respect to every recycling cycle, showing the surface roughness differentiation over consecutive recycling cycles (Figure 10c).

#### 3.1.2. TX1501 with 7.3%cCFs

Similarly to PA12 with 8.6%cCFs and 7.5%MNPs, the printability tests on TX1501 with 7.3%cCFs were performed, applying the same printing parameters values within the printable ranges of the material to all the examined cycles. Table 6 displays the selected printing parameters values, where, again, the printing speed values in Test04 and Test06 were defined independently for each material cycle according to the optimal parameter derived from Test03 and Test04, respectively. Corresponding to the methodology followed for PA12 with 8.6%cCFs and 7.5%MNPs, the increment step between the printing speed tested values for TX1501 with 7.3%cCFs was set 10 mm/s and 2.5 mm/s for Test04 and Tes06, respectively. Table 7 presents the printing speed values applied in Tests04 and Tests06 to all cycles of TX1501 with 7.3%cCFs assessed.

**Figure A6:**
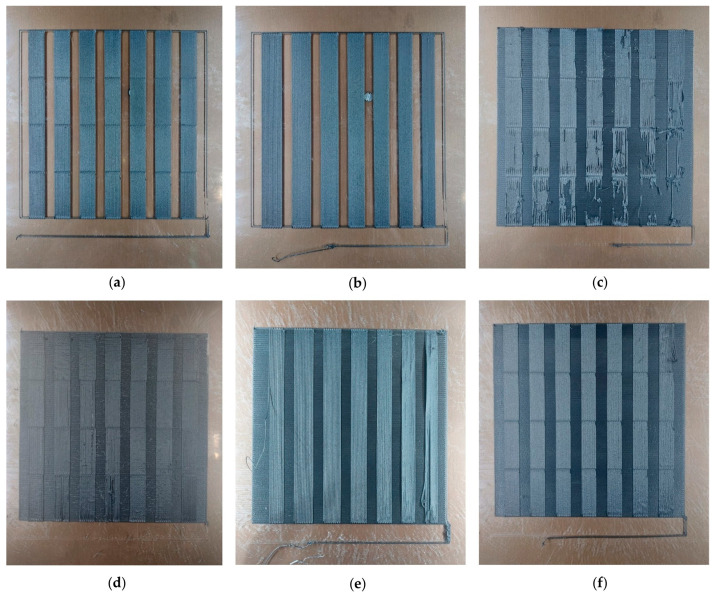
Printability samples of TX1501 with 7.3%cCFs (Control Material): (**a**) Test01; (**b**) Test02; (**c**) Test03; (**d**) Test04; (**e**) Test05; (**f**) Test06.

**Figure A7:**
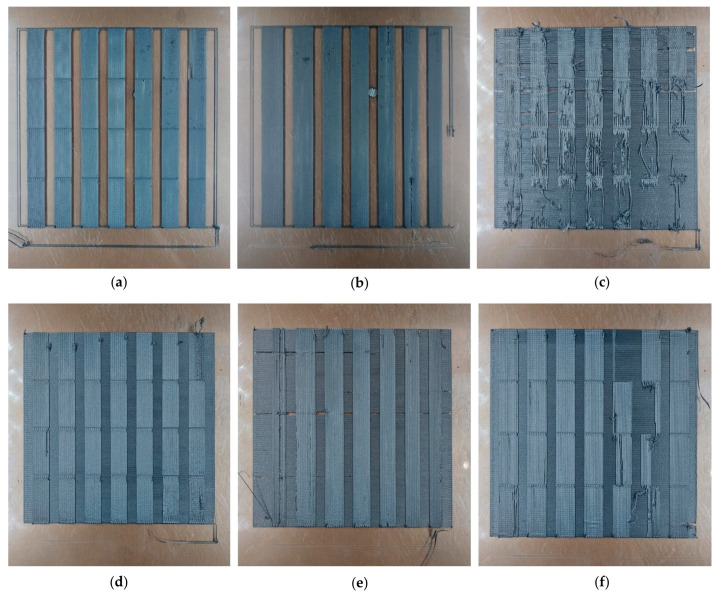
Printability samples of TX1501 with 7.3%cCFs (1st Cycle): (**a**) Test01; (**b**) Test02; (**c**) Test03; (**d**) Test04; (**e**) Test05; (**f**) Test06.

**Figure A8:**
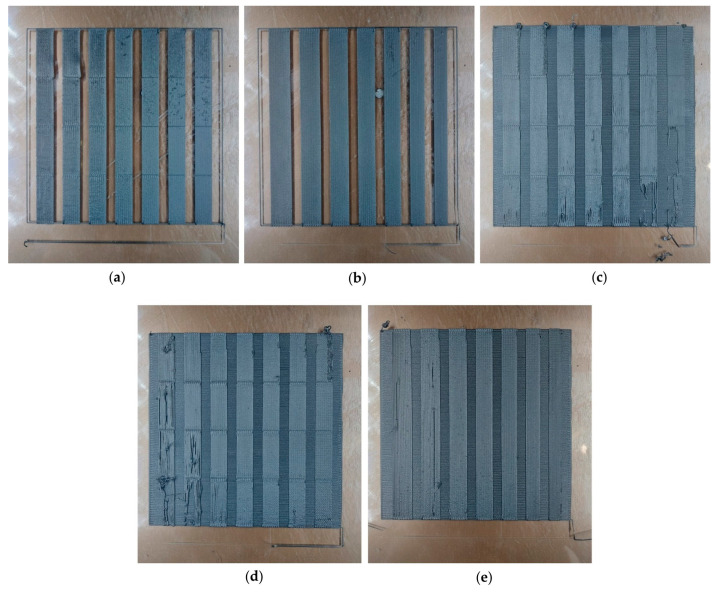
Printability samples of TX1501 with 7.3%cCFs (5th Cycle): (**a**) Test01; (**b**) Test02; (**c**) Test03; (**d**) Test04; (**e**) Test05.

**Figure A9:**
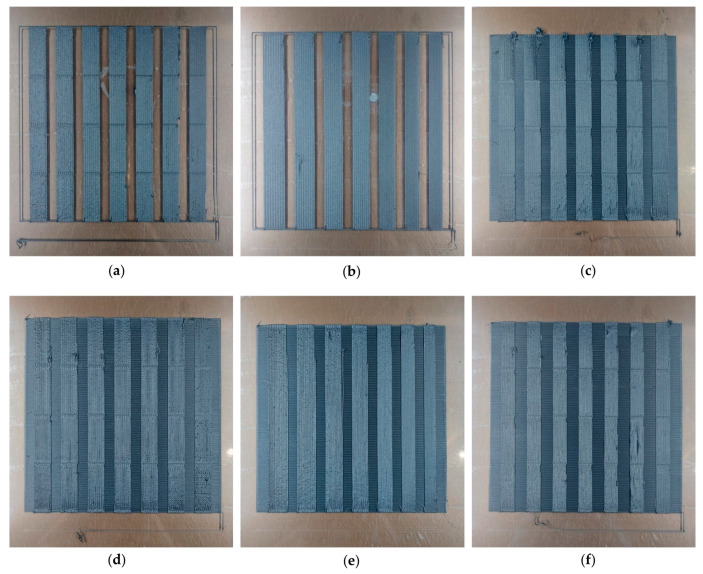
Printability samples of TX1501 with 7.3%cCFs (10th Cycle): (**a**) Test01; (**b**) Test02; (**c**) Test03; (**d**) Test04; (**e**) Test05; (**f**) Test06.

Figure A6, Figure A7, Figure A8 and Figure A9 present the printability samples produced with the Control Material, 1st Cycle, 5th Cycle and 10th Cycle of TX1501 with 7.3%cCFs. As can be deduced from Figure A6, the Control Material of TX1501 with 7.3%cCFs presented satisfactory printability for printing speed values lower than 40 mm/s and extrusion temperatures over 265 °C (Figure A6c). The 1st Cycle of TX1501 with 7.3%cCFs exhibited limited printability, and each test had to be printed multiple times in order to obtain completed test samples since nozzle clogging repeatedly occurred during the printing procedure, which was highly associated with the filament diameter fluctuation and the blockage of the material extrusion route. Also, as observed from Figure A7c, the material exhibits adequate printability only with printing speed values lower than 20 mm/s. Moreover, concerning Test06, nozzle clogging occurred again during the printing of the second and third columns, but due to material shortage, the tests could not be reperformed. The 5th Cycle of TX1501 with 7.3%cCFs presented satisfactory printability, except for temperature values below 255 °C and printing speed values greater than 60 mm/s (Figure A8c). Figure A8d shows that nozzle clogging occurs for layer track height values greater than 0.45 mm. Due to material inefficiency, Test06, concerning extrusion multiplier and printing speed fine-tuning, could not be performed, and therefore, the optimal printing speed established is the printing speed selected from Test04 (Table 7). Finally, the 10th Cycle of the material exhibited great printability in the tested printing parameter ranges, and in contrast to the three previously tested material cycles, it was printable in every examined extrusion temperature value even when utilizing high printing speed values, such as 80 mm/s (Figure A9c).

**Figure 11:**
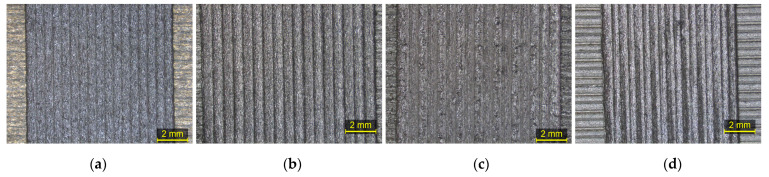
Stereoscope images of optimum regions: (**a**) Control Material (Test06); (**b**) 1st Cycle (Test06); (**c**) 5th Cycle (Test05); (**d**) 10th Cycle (Test06).

Table 8 and Figure 11 display the optimal parameters derived from the printability assessment and the stereoscope images that correspond to the optimum regions of TX1501 with 7.3%cCFs, namely the Control Material, 1st Cycle, 5th Cycle, and 10th Cycle.

**Figure 12:**
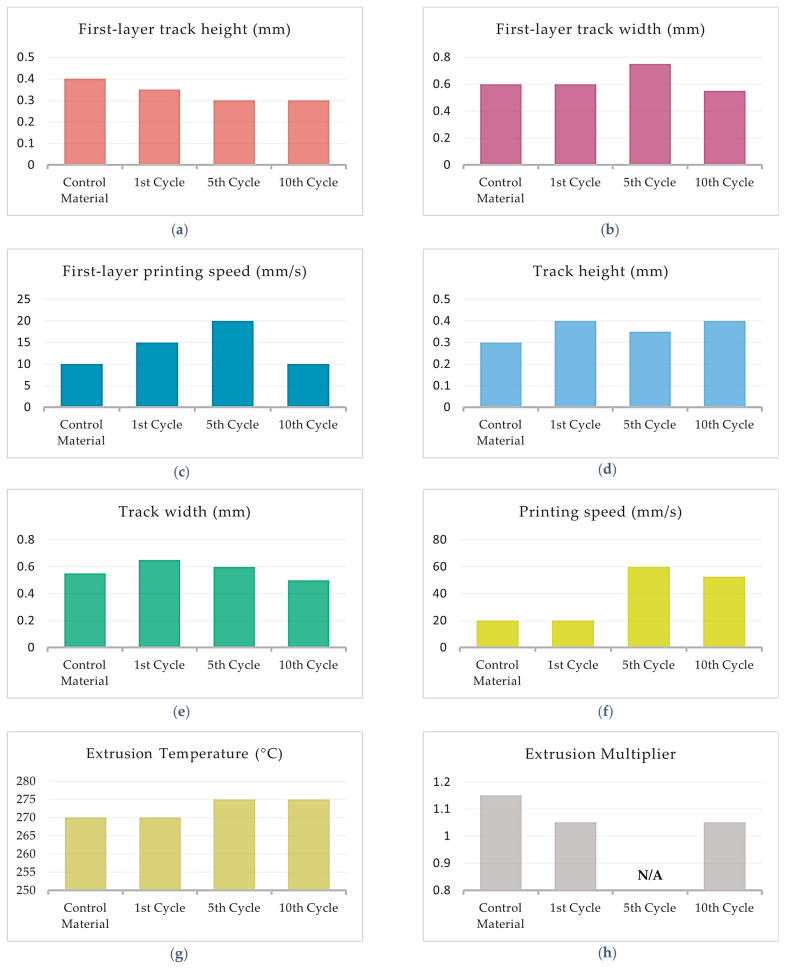
Optimal printing parameter value fluctuation of TX1501 with 7.3%cCFs through recycling cycles: (**a**) First-layer track height; (**b**) First-layer track width; (**c**) First-layer printing speed; (**d**) Track height; (**e**) Track width; (**f**) Printing speed; (**g**) Extrusion temperature; (**h**) Extrusion multiplier.

Figure 12a–h presents the fluctuation of the established optimal printing parameters values through the recycling cycles of TX1501 with 7.3%cCFs for the first layer and the main printing layers, respectively.

Figure 12a shows that the optimal value of the First-layer track height exhibits a slow decrease through the recycling cycles from 0.4 mm to 0.3 mm, while Figure 12b,c shows that the First-layer track width and printing speed optimal values exhibit a fluctuation through the recycling cycles with maximum deviation from the corresponding Control Material values, i.e., 25% and 100%, respectively.

Figure 12d,e shows that the optimal values of track height and track width for TX1501 with 7.3%cCFs fluctuate between the ranges of 0.3–0.4 mm and 0.50–0.65 mm, respectively, through the recycling cycles. Figure 12f indicates that the optimal value of the printing speed is increased significantly since for the Control Material and 1st Cycle, it was established as 20 mm/s, while for the 5th and 10th Cycles, the corresponding values were almost three times greater, at 60 mm/s and 52.5 mm/s, respectively. From Figure 12g, it can be inferred that through the recycling cycles, the optimal value of extrusion temperature is slightly increased from 270 °C, for the Control Material and 1st Cycle, to 275 °C, for the 5th and 10th Cycles. Finally, regarding the optimal extrusion multiplier, due to the material shortage of the 5th Cycle mentioned, Figure 12h displays only the values established for the Control Material, 1st Cycle and 10th Cycle, which, as can be deduced the respective values of the recycled filaments (1st and 10th Cycles), exhibit an 8.7% decrease compared to the Control Material.

**Figure 13:**
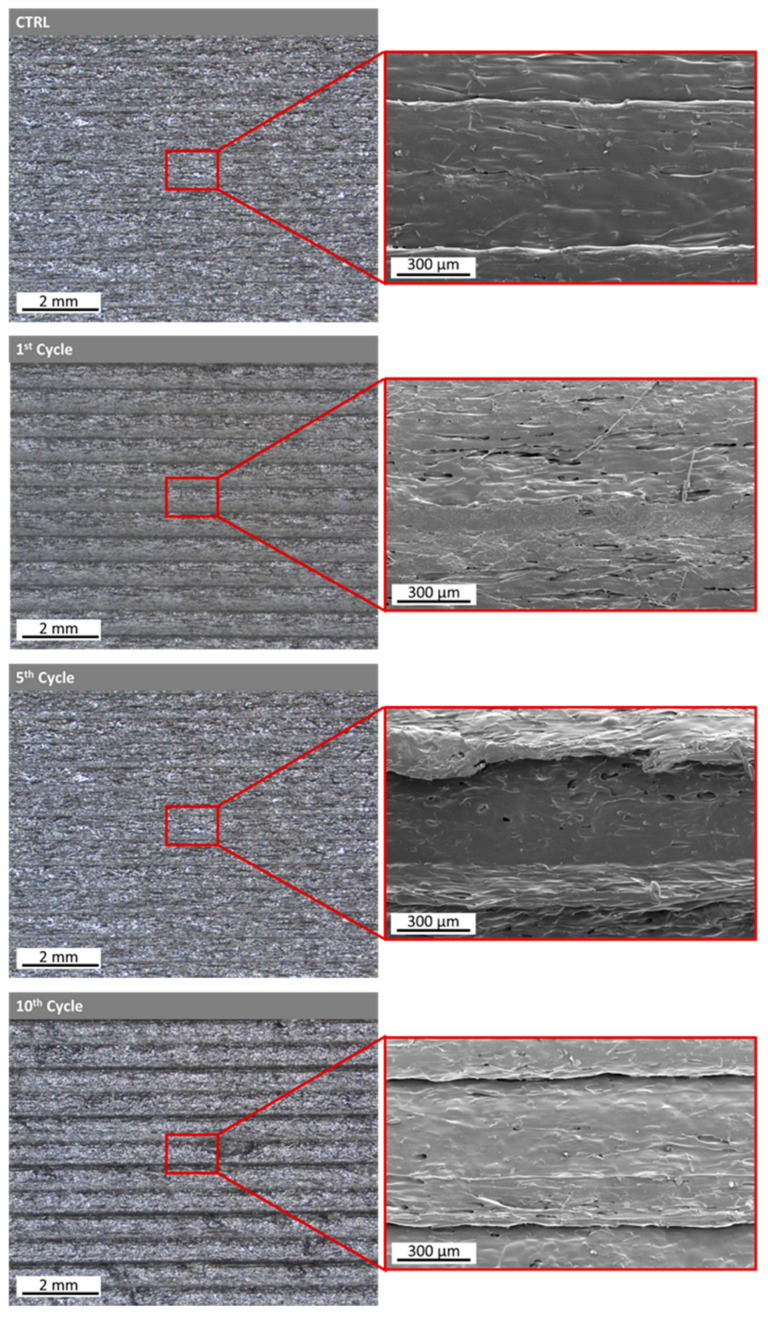
Stereoscope and SEM images for surface assessment after the first, fifth and tenth recycling cycles of nanocomposite TX1501 filament. Surface comparison using the control’s optimized printing parameters.

**Figure 14:**
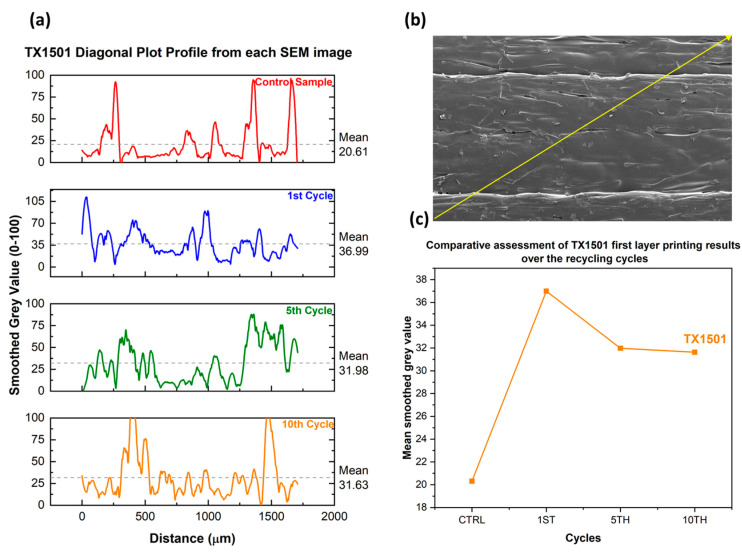
(**a**) Smoothed plot profiles from the grey values obtained alongside the diagonal line from each TX1501 SEM image. (**b**) Indicative diagonal line from which the grey values were obtained. (**c**) The evolution of the mean grey values from each recycling cycle.

A surface morphology comparison using the optimized Control Material printing parameters in every recycling cycle was conducted by employing SEM morphology assessment (Figure 13). Image processing utilizing ImageJ was performed, and surface plot profiles were generated for every recycling cycle to assess the effect of the recycling cycles (Figure 14a) to the surface roughness alongside the yellow diagonal line (Figure 14b). Then, the mean grey values were plotted in respect to every recycling cycle, showing the surface roughness differentiation over consecutive recycling cycles (Figure 14c).

### 3.2. In Vitro Toxicity of the Recycled Materials

#### 3.2.1. Cell Viability upon Exposure to the Recycled Materials

**Figure 15:**
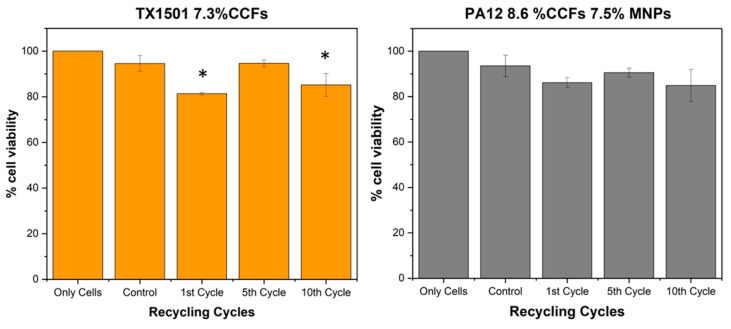
Cell viability assessment upon exposure to the recycled materials for 48 h using MTT assay. (*) corresponds to a *p*-value > 0.05.

MTT assay was conducted to evaluate the viability of the human skin fibroblast cell line (Hs27) upon direct contact with the recycled materials after 48 h. As shown in Figure 15, there is a significant reduction in the cell viability upon exposure to the TX1501 samples after the 1st and 10th recycling cycles by performing one-way Analysis of Variance (ANOVA) or T test. Although a slight reduction is also observed after the 5th Cycle, it does not seem to be statistically significant; it is likely that more measurements would clarify this result. PA12 samples seem to not affect the viability of the cells in any of the recycling cycles.

#### 3.2.2. Skin-Sensitizing Potential of the Recycled Materials

The aseptic samples were soaked in complete cell medium overnight prior to introducing cells. After exposing the skin cells to the recycled materials for 48 h, the release of the proinflammatory cytokines IL-18 and TNF-α, which serve as indicators of potential skin sensitization, was measured in the cell culture medium using ELISA, following the manufacturer’s instructions.

**Figure 16:**
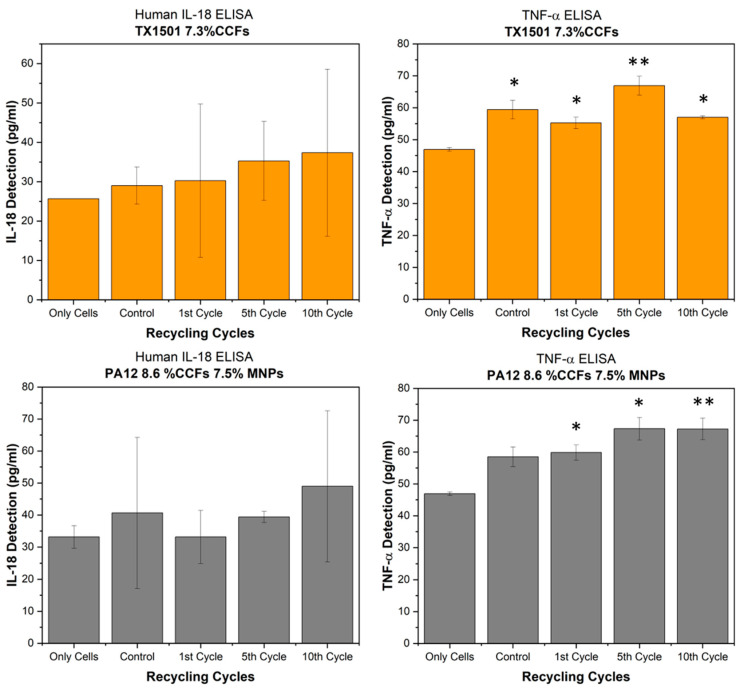
Skin-sensitizing potential of the recycled materials. Detection of human proinflammatory cytokines (IL-18, left; TNF-α, right) was evaluated via ELISA. (*) corresponds to a *p*-value > 0.05 and (**) to a *p*-value> 0.01.

To determine the concentration of IL-18 and TNF-α, calibration curves were generated and analyzed. The obtained concentrations (pg/mL) were calibrated using the equation of the calibration curve, and the average concentration for each sample was compared with the respective control. The negative control represented cells without any material. Materials prior to recycling were used as control materials. The statistical analysis of the results involved performing a one-way Analysis of Variance (ANOVA) test. *p*-values greater than 0.05 were considered not statistically significant. As shown in the graphs (Figure 16), none of the materials seem to significantly induce the release of the cytokine IL-18. On the other hand, both TX1501 and PA12 samples significantly induce the release of TNF-α cytokines in the cell culture media, leading to the conclusion that the recycling cycles of both materials affect skin cells in vitro and might possibly affect their skin-sensitizing potential.

## 4. Discussion

This paper presented a study on the assessment of the discrete recycling cycles of custom filaments made of PA12 with 8.6%cCFs and 7.5%MNPs and TX1501 with 7.3%cCFs developed for the FFF manufacturing of wearable devices. The initially developed materials and the 1st, 5th and 10th Cycles of recycling were evaluated in terms of parameter optimization, printing surface quality performance, process parameters fluctuation and cytotoxicity. 

A stereoscope was employed for the surface inspection, overflow and underflow defect detection and quantification, material flow uniformity evaluation and, finally, the optimal parameter determination of each recycling cycle tested. The results indicated that TX1501 with 7.3%cCFs presented adequate printability performance even after 10 cycles of recycling, generating parts with limited defects, acceptable material uniformity and adequate interlayer adhesion. On the other hand, PA12 with 8.6%cCFs and 7.5%MNPs was printable only as a Control Material and at the 1st and 5th Cycles of recycling. The study of the optimal printing parameters through the recycling cycles of both materials illustrates that slight or no deviations are exhibited in the extrusion temperature and extrusion multiplier values, while the optimal printing speed in the main layers is increased. Furthermore, the established optimal values of the other printing parameters are of great interest, of which fluctuations from the respective values of the Control Material are exhibited.

The second stage of printability assessment and surface evaluation was realized with SEM comparing part of the regions of every recycling cycle printed with the Control Material optimal parameters. The obtained images and surface analysis dictate that both TX1501 and PA12 nanocomposite filaments result in greater roughness compared to the Control Material after one recycling cycle. After the 5th recycling cycle, the roughness of both materials decreases. By completing 10 recycling cycles, the roughness of the PA12 increases in contrast to TX1501, which decreases. However, the difference in the roughness values (denoted by the mean smoothed grey values) between the 5th and 10th recycling cycles is relatively small and shows a trend towards converging around toward a constant value. This could be explained due to the fact that the polymeric matrix has been already thermally degraded from the 1st Cycle, and no effect on the surface roughness occurs when recycling cycles increase. Furthermore, TX1501 presents greater roughness than PA12 due to the higher process temperature and higher cCF loading (Figure 14).

Copolyesters and polyamides are hygroscopic materials [33], although by adding fillers, such as glass or carbon fibers, the hygroscopicity can be reduced [34]. The cCF concentration inside the PA12 nanocomposite filament is greater than that inside TX1501. Thus, there is no need for higher temperatures for the nanocomposite PA12 to fully dry and sufficiently extrude from the nozzle. Moreover, after 10 recycling cycles, PA12 was not printable. This can be explained by the extended degradation of the polymeric matrix, leading to a lower molecular weight and reduced melt viscosity. This leads to an increase in the cCFS and MNP mobility inside the matrix during extrusion. Hence, extrusion issues occurred, such as filament clogging, while insufficient bed adhesion was investigated due to the compromised filament quality.

Furthermore, in vitro studies of the recycled materials were performed in order to reveal possible adverse effects on the skin cells after direct contact. The results suggest that the TX1501 recycled material negatively affects the viability of Hs27 human skin fibroblast cells, particularly after the 1st and 10th recycling cycles. Conversely, the PA12 samples did not demonstrate any significant influence on cell viability. These findings highlight the importance of considering the effects of recycled materials on cell viability when evaluating their potential applications in various contexts. Further research is warranted to fully understand the mechanisms underlying these observations and to explore potential strategies for mitigating any adverse effects associated with recycled materials. Additionally, none of the materials significantly induced the release of the cytokine IL-18. However, both the TX1501 and PA12 samples significantly induced the release of TNF-α cytokines into the cell culture media. These results emphasize the importance of considering the potential inflammatory responses of skin cells to recycled materials. The induction of TNF-α release suggests the possible activation of the immune system and highlights the need for further investigation into the potential skin-sensitization effects of these recycled materials.

The printability assessment in the present study dealt with the inspection of specific defects type on the printed samples, namely overfill, underfill defects and low material uniformity. Moreover, since the quantity of the developed composite material batches was specific, the printing repeat of failed printability tests of even the implementation of the final tests in some cases was restricted due to material shortage, in particular, recycling cycles. In regard to the SEM examination, the evaluation of every 3D-printed region produced within the first stage of printability study would further assist decision making towards the optimized printing parameters. In addition, to better understand the impact of the consecutive recycling cycles on the materials’ printability and the overall performance of the final 3D-printed parts, Fourier Transform Infrared spectroscopy (FTIR), Differential Scanning Calorimetry (DSC) mechanical testing, and Force Atomic Microscopy (AFM) could be included in the characterization process.

## 5. Conclusions

The first stage of printability evaluation performed using a stereoscope facilitated the printability examination of each recycling cycle and illustrated the fluctuation of the optimal printing parameters values, indicating for both TX1501 with 7.3%cCFs and PA12 with 8.6%cCFs and 7.5%MNPs that minor deviations are presented in the optimal extrusion temperature, while the value of the main-layer printing speed is increased. In the second stage of printability assessment, SEM analysis and surface roughness were performed to assess a part of the 3D-printed regions of every recycling cycle utilizing the same printing parameters and the optimal First-layer track height and printing speed established for the Control Material. The results showed that the mean roughness values of the 1st Cycle increased, while in the last recycling cycles, they decreased, and that the difference between the latter cycles is relatively small and shows a trend towards converging toward a constant value. Last but not least, in vitro studies provided information on possible adverse effects of the materials after recycling by using human skin cells. The results highlight the importance of considering the effects of the recycled materials when evaluating their potential applications in medical device development, such as wearables and/or other products that come in direct contact with a human tissue (e.g., skin).

More specifically, the mains conclusion derived from the first stage of printability assessment through stereoscopic observation can be summarized as follows:

- TX1501 with 7.3%cCFs exhibited consistent printability performance even after 10 cycles of recycling, producing parts with limited defects, acceptable material uniformity, and interlayer adhesion;
- PA12 with 8.6%cCFs and 7.5%MNPs was printable only as a Control Material and in the 1st and 5th Cycles of recycling;
- Optimal printing parameters, specifically extrusion temperature, extrusion multiplier, and printing speed, exhibited slight deviations or remained consistent throughout the recycling cycles.

Furthermore, concerning the surface evaluation via SEM, the following was indicated:

- Both TX1501 and PA12 nanocomposite filaments displayed higher roughness compared to the Control Material after the 1st recycling cycle, with subsequent variations in roughness levels as recycling progressed;
- The difference in roughness values between 5th and 10th recycling cycles converged toward a constant value, implying minimal changes in surface roughness due to thermal degradation of the polymeric matrix;
- TX1501 presented higher roughness than PA12, which was attributed to the higher process temperature and cCF loading.

Finally, in vitro studies on the tested recycled materials implied the following:

- TX1501 negatively affected the viability of human skin fibroblast cells, particularly after the 1st and 10th recycling cycles, whereas PA12 did not significantly influence cell viability;
- The importance of considering cell viability and inflammatory responses in evaluating recycled materials’ potential applications was highlighted.

A limitation of this study is the reliance on manual observation for defect inspection and the decision-making process. To address this, we propose automation through image analysis and machine learning as a potential avenue for further research. By automating these processes, we can enhance objectivity and accuracy when assessing defects and optimizing printing parameters [35]. However, the purpose of the current study is to establish a straightforward workflow for material evaluation and the printing parameter optimization of the different recycling cycles of composite filaments based on material performance, as observed through stereoscope, as an efficient and cost-effective alternative to extended material testing and characterization methods.

## Figures and Tables

**Table 1:** Parameters evaluated at each printability test.

Test No	Printing Parameters Examined
1	First-layer track height vs. First-layer printing speed
2	First-layer track width
3	Extrusion temperature vs. Printing speed
4	Track height vs. Printing speed
5	Track width
6	Extrusion multiplier vs. Printing speed

**Table 2:** Printing parameters applied in Test01 samples of PA12 with 8.6%cCFs and 7.5%MNPs and TX1501 with 7.3%cCFs.

Printing Parameter	PA12 with 8.6%cCFs and 7.5%MNPs	TX1501 with 7.3%cCFs
Extrusion temperature	230 °C	260 °C
Bed temperature	100 °C	60 °C
First-layer track width	0.6 mm	0.6 mm
Extrusion multiplier	1.00	1.00

**Table 3:** Values of the tested printing parameters for PA12 with 8.6%cCFs and 7.5%MNPs.

Test No	Printing Parameter	Values Tested						
Test01	First-layer track height (mm)	0.2	0.25	0.3	0.35	0.4	0.45	0.5
	First-layer printing speed (mm/s)	5	10	15	20			
Test02	First-layer track width (mm)	0.38	0.43	0.48	0.53	0.58	0.63	0.68
Test03	Extrusion temperature (°C)	210	220	230	240	250	260	270
	Printing speed (mm/s)	15	30	45	60			
Test04	Track height (mm)	0.2	0.25	0.3	0.35	0.4	0.45	0.5
	Printing speed (mm/s)	Speed1	Speed2	Speed3	Speed4			
Test05	Track width (mm)	0.45	0.5	0.55	0.6	0.65	0.7	0.75
Test06	Extrusion multiplier	0.9	0.95	1	1.05	1.1	1.15	1.2
	Printing speed (mm/s)	Speed1′	Speed2′	Speed3′	Speed4′			

**Table 4:** Printing speed values tested in Test04 and Test06 for PA12 with 8.6%cCFs and 7.5%MNPs (Control Material, 1st Cycle and 5th Cycle).

Recycling Cycles	Test No	Printing Speed Values Tested (mm/s)				Selected Value (mm/s)
Control Material	Test04	15	20	25	30	20
	Test06	15	17.5	20	22.5	17.5
1st Cycle	Test04	25	30	35	40	25
	Test06	22.5	25	27.5	30	22.5
5th Cycle	Test04	25	30	35	40	35
	Test06	30	32.5	35	37.5	30

**Table 5:** Optimal printing parameters of PA12 with 8.6%cCFs and 7.5%MNPs (Control Material, 1st Cycle, 5th Cycle and 10th Cycle).

Printing Parameter	Control Material	1st Cycle	5th Cycle	10th Cycle
First-layer track height	0.25 mm	0.4 mm	0.25 mm	0.4 mm
First-layer printing speed	10 mm/s	5 mm/s	15 mm/s	10 mm/s
First-layer track width	0.48 mm	0.53 mm	0.48 mm	0.48 mm
Extrusion Temperature	250 °C	250 °C	250 °C	-
Track height	0.40 mm	0.3 mm	0.3 mm	-
Track width	0.50 mm	0.55 mm	0.55 mm	-
Printing speed	17.5 mm/s	22.5 mm/s	30 mm	-
Extrusion multiplier	1.15	1.15	1.1	-

**Table 6:** Values of the tested printing parameters for TX1501 with 7.3%cCFs.

Test No	Printing Parameter	Values Tested						
Test01	First-layer track height (mm)	0.2	0.25	0.3	0.35	0.4	0.45	0.5
	First-layer printing speed (mm/s)	5	10	15	20			
Test02	First-layer track width (mm)	0.45	0.5	0.55	0.6	0.65	0.7	0.75
Test03	Extrusion temperature (°C)	250	255	260	265	270	275	280
	Printing speed (mm/s)	20	40	60	80			
Test04	Track height (mm)	0.2	0.25	0.3	0.35	0.4	0.45	0.5
	Printing speed (mm/s)	Speed1	Speed2	Speed3	Speed4			
Test05	Track width (mm)	0.45	0.5	0.55	0.6	0.65	0.7	0.75
Test06	Extrusion multiplier	0.85	0.9	0.95	1	1.05	1.1	1.15
	Printing speed (mm/s)	Speed1′	Speed2′	Speed3′	Speed4′			

**Table 7:** Printing speed values tested in Test04 and Test06 for TX1501 with 7.3%cCFs (Control Material, 1st Cycle, 5th Cycle and 10th Cycle).

Recycling Cycles	Test No	Printing Speed Values Tested (mm/s)				Selected Value (mm/s)
Control Material	Test04	10	20	30	40	20
	Test06	17.5	20	22.5	25	20
1st Cycle	Test04	10	20	30	40	20
	Test06	17.5	20	22.5	25	20
5th Cycle	Test04	40	50	60	70	60
10th Cycle	Test04	40	50	60	70	50
	Test06	47.5	50	52.5	55	52.5

**Table 8:** Optimal printing parameters of TX1501 with 7.3%cCFs (Control Material, 1st Cycle, 5th Cycle and 10th Cycle).

Printing Parameter	Control Material	1st Cycle	5th Cycle	10th Cycle
First-layer track height	0.4 mm	0.35 mm	0.3 mm	0.3 mm
First-layer printing speed	10 mm/s	15 mm/s	20 mm/s	10 mm/s
First-layer track width	0.6 mm	0.6 mm	0.75 mm	0.55 mm
Extrusion temperature	270 °C	270 °C	275 °C	275 °C
Track height	0.3 mm	0.4 mm	0.35 mm	0.4 mm
Track width	0.55 mm	0.65 mm	0.6 mm	0.5 mm
Printing speed	20 mm/s	20 mm/s	60 mm/s	52.5 mm/s
Extrusion multiplier	1.15	1.05	-	1.05

## Data Availability

Data available upon request.

## Abbreviations

**Abbreviation**	**Meaning**
AFM	Atomic Force Microscopy
AM	Additive Manufacturing
ANOVA	Analysis of Variance
CFs	Carbon Fibers
cCFs	Chopped Carbon Fibers
vCFs	Virgin Carbon Fibers
rCFs	Recycled Carbon fibers
CO2	Carbon Dioxide
DMEM	Dulbecco’s Modified Eagle Medium
DRAM	Distributed Recycling via Additive Manufacturing
DSC	Differential Scanning Calorimetry
ELISA	Enzyme-linked Immunosorbent Assay
FBS	Fetal Bovine Serum
FDM	Fused Deposition Modeling
FFF	Fused Filament Fabrication
FTIRGFRPPrGFs	Fourier Transform Infrared SpectroscopyGlass-fiber-reinforced PolypropyleneRecycled Glass Fiber
HDPE	High-density Polyethylene
IL	Interleukin
ISO	International Organization for Standardization
LCA	Life Cycle Assessment
MNP	Magnetic Nanoparticles
PA12	Polyamide 12
PBS	Phosphate-buffered Saline
PET	Poly (ethylene terephthalate)
PET-G	Polyethylene Terephthalate Glycol
PLA	Polylactic Acid
PP	Polypropylene
SEM	Scanning Electron Microscopy
STP	Standard Temperature and presSsure
TNF	Tumor Necrosis Factor
TX1501	Tritan Copolyester TX1501
USA	United States of America
